# When to Suspect DRUJ's Instability in Children? Case Report of a Rare Presentation of Distal Forearm Fractures

**DOI:** 10.1055/s-0042-1748317

**Published:** 2022-06-09

**Authors:** Claire-Anne Saugy, Aline Bourgeois Bregou

**Affiliations:** 1 Service d'Orthopédie, Hôpital de zone de Morges, Morges, Switzerland; 2 Unité Pédiatrique de Chirurgie Orthopédique et Traumatologique, CHUV, Lausanne, Vaud, Switzerland

**Keywords:** Forearm fracture, Children, distal radioulnar joint, Galeazzi-equivalent injury

## Abstract

Pediatric displaced distal metaphyseal ulnar fractures and distal radial buckle fractures are common. However, to the best of our knowledge, their association has never been specifically reported. Thus, classification and management of this pattern remain challenging especially in young children. Distal radioulnar joint (DRUJ)'s assessment is difficult. A Galeazzi-equivalent injury should be suspected. We report the case of a 2-year-old boy who presented the above-mentioned association of forearm fractures and compare our management with actual recommendations. We would recommend a low-suspicion threshold for DRUJ's instability in young children presenting with displaced distal metaphyseal ulnar fracture associated with distal radial buckle fracture. That suspicion should raise the necessity of appropriate treatment and follow-up.

## Introduction

Distal forearm fractures are the most common fractures among children. 1 2 Several injury patterns are possible: torus/buckle, greenstick, complete, displaced or undisplaced metaphyseal fractures, physeal fractures, and Galeazzi-equivalent injuries. 3 4 The radius is predominantly concerned. 2 Depending on the residual growth and the remodeling capacity of the growing bone, treatment modalities include cast immobilization with or without closed reduction, and closed or open reduction with fixation in the most severe injuries. 1 However, to the best of our knowledge, the following pattern has never been described: displaced metaphyseal ulnar fracture associated with distal radial buckle fracture. Thus, classification and management of this pattern remain challenging. Stability of distal radioulnar joint (DRUJ) must be maintained.

## Case Report

**Fig. 1:**
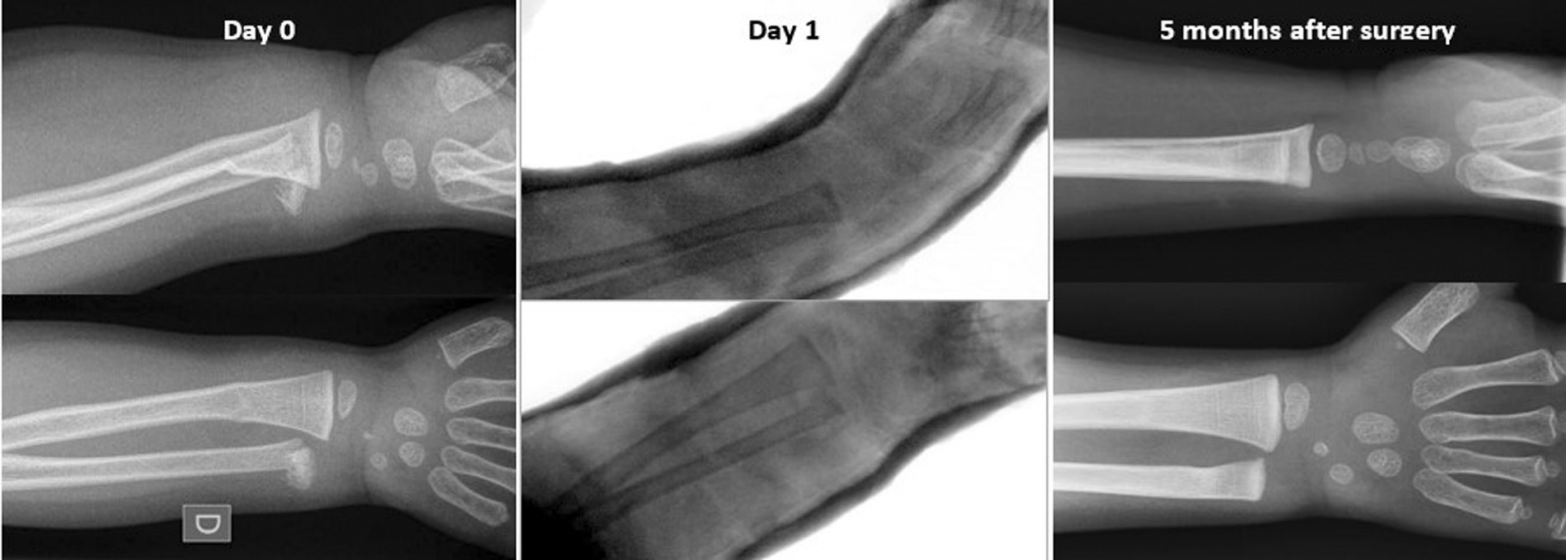
Serial X-rays of the patient.

We report the case of a 2-year-old boy who fell from the height of 30 cm and sustained a right forearm trauma. No adult witnessed the fall. Initial workup showed a painful deformation of the right wrist without neurovascular disorder and skin lesion. X-rays revealed a displaced metaphyseal ulnar fracture with a 30-degree posterior tilt associated with a distal radial buckle fracture ( Fig. 1 ). Pediatric orthopaedic surgeons performed a closed reduction under general anesthesia and an above elbow plaster cast immobilization, with small palmar flexion and forearm neutral position ( Fig. 1 ). It remained for 4 weeks and was then replaced by a wrist brace for 4 more weeks.

Clinical and radiological evolution were good ( Fig. 1 ). Two months after surgery, the child had no residual pain, no range of motion limitation, and no DRUJ's instability. Activities with risk of falling were then allowed. Follow-up was discontinued 5 months after surgery.

## Discussion

Distal radius fractures are very common during childhood. 5 6 They mostly result from a fall onto an outstretched hand. 6 And they are frequently associated with ulnar fractures (diaphysis, metaphysis, or ulnar styloid process). 2 6 Isolated ulnar fracture is uncommon. 2

One specific injury pattern combines distal radius fracture with DRUJ's disruption. It includes true ulnar dislocation (called Galeazzi injury) or ulnar epiphyseal avulsion associated with displacement (called Galeazzi-equivalent injury). 3 5 Because of the relative weakness of bone in comparison with ligaments in skeletally immature children, Galeazzi-equivalent injury occurs exclusively in this population and predominates among young teenagers. 3 4 5 7 However, this pattern seems infrequent although probably underestimated. 7 8 The initial clinical assessment of the DRUJ could indeed be impossible in young children suffering from pain. One study showed that displaced Salter Harris 2 fractures of the radius and nonphyseal distal radius fractures were the most common injuries associated with DRUJ's instability in a population of 85 patients (mean age at trauma: 14 years, range: 6.7–17.8). Time between trauma and DRUJ's instability diagnosis ranged from 0 to 18 years (mean: 3 years). 9

Galeazzi-equivalent injuries are classified according to the Letts and Rowhani classification 3 10 ( Table 1 ).

**Table 1:** Letts and Rowhani classification 3 10

Type		Description
**A**		Fracture of the radius at the junction of the middle and distal thirds +
	**1**	Dorsal dislocation of the ulna
	**2**	Epiphyseal fracture of the distal ulna with dorsal displacement of ulnar metaphysis
**B**		Fracture of the distal third of the radius +
	**1**	Dorsal dislocation of the ulna
	**2**	Epiphyseal fracture of the distal ulna with dorsal displacement of ulnar metaphysis
**C**		Greenstick fracture of the radius with dorsal bowing +
	**1**	Dorsal dislocation of distal ulna
	**2**	Epiphyseal fracture of distal ulna with displacement of ulnar metaphysis
**D**		Fracture of distal radius with volar bowing +
	**1**	Volar dislocation of the ulna
	**2**	Epiphyseal fracture of distal ulna with volar displacement of ulnar metaphysis

Unlike in adults, most of the Galeazzi-equivalent injuries in children are treated by closed reduction and immobilization in an above elbow plaster cast for 4 to 6 weeks. 3 7 11 The forearm should be placed in full supination. 10 11 Major residual instability, irreducible fracture, and dislocation are treated with open reduction. 4 Reported results are good. 7 12 However, there is a paucity of data on long-term results. One retrospective study including 10 children (mean age: 13.7 years, range: 11–16) with a mean follow-up of 6 years showed ulnar length discrepancy, bony deformation, or joint incongruence in the majority of the patients. 12 Misdiagnosed injuries or improper treatment could also compromise the DRUJ' stability, the wrist and forearm range of motion, and generate chronical pain.

To the best of our knowledge, our patient's fractures pattern has never been specifically described in the literature and is unusual for his age. It combines a displaced distal metaphyseal ulnar fracture and a distal radial buckle fracture. Each of these fractures is quite common, but their association is infrequent. Our initial differential diagnosis included the Galeazzi-equivalent injury versus “simple” metaphyseal fractures without DRUJ's instability. The DRUJ' stability could not be assessed in the emergency unit because of pain and swelling. Initial X-rays showed a certain displacement between both bones. However, no age-specific diagnostic radiographic measurements are described in literature for DRUJ's instability. An ulnar epiphyseal injury could also not be excluded because of the nonossified nature of the epiphysis in young children.

Our treatment included closed reduction under general anesthesia and immobilization in an above elbow plaster cast with the forearm in neutral alignment. Immobilization remained for a total of 8 weeks. Regarding the healing ability of young patients, we could question the length of our immobilization. Prognosis of radial buckle fracture is excellent because of the lack of significant displacement and retained residual stability. 13 It allows short immobilization (2–4 weeks). 14 Displaced metaphyseal fractures in children usually need longer immobilization (4–6 weeks). 13 In up to 30% of complete radial metaphyseal fractures, loss of reduction occurs after closed reduction and immobilization. 1 13 15 However, malunion, nonunion, and refracture are rarely observed. 1 No such data are available for complete ulnar metaphyseal fractures.

The injury in our patient healed without sequela. However, we recommend a low-suspicion threshold for DRUJ's instability in young children presenting with displaced distal metaphyseal ulnar fracture associated with distal radial buckle fracture. That suspicion should raise the necessity of appropriate treatment and follow-up. Delay between trauma and DRUJ's instability diagnosis can indeed be long.

## Notes

New Insights & the Importance for the Pediatric Surgeon DRUJ's assessment is required in young children presenting unusual distal forearm fractures (e.g., displaced distal metaphyseal ulnar fracture associated with distal radial buckle fracture). It may impact treatment and follow-up.
